# COVID-19 Pneumonia in Asymptomatic Trauma Patients; Report of 8 Cases

**Published:** 2020-04-06

**Authors:** Majid Samsami, Javad Zebarjadi Bagherpour, Behzad Nematihonar, Hamed Tahmasbi

**Affiliations:** 1 Department of General Surgery, Imam Hossein Hospital, Shahid Beheshti University of Medical Sciences, Tehran, Iran

**Keywords:** COVID-19, Pneumonia, injuries

## Abstract

We are currently involved in the novel coronavirus 2019 (COVID-19) pandemic. A considerable number of COVID-19 infected cases are asymptomatic but they can transmit the disease to others, especially healthcare workers. In this study, we reported 8 incidentally detected cases of COVID-19 pneumonia in chest computed tomography (CT) scan of patients referred to emergency department following multiple trauma without any respiratory symptoms.

## Introduction:

The global incidence of novel coronavirus 2019 (COVID-19) that involves the lower respiratory tract (pneumonia) continues to rise since December 2019 (1). The specific source and the exact primary mode of transmission of 2019-nCoV to humans remain unknown. The clinical features and laboratory and radiological abnormalities of COVID-19 infections are not specific and are similar to other respiratory tract infections (2). It is now evident that most cases of COVID-19 disease develop mild respiratory and constitutional symptoms such as fever, cough, dyspnea, myalgia, and fatigue (3). A considerable number of COVID-19 infected cases are asymptomatic but they can transmit the disease to others, especially healthcare workers (4). In the study performed by Khazaee et al. (5), several patients had incidental evidence of COVID-19 infection on chest CT scans obtained for trauma management. In another study, Hu et al. (6), described clinical features of 24 asymptomatic patients on their study, five cases (20.8%) developed symptoms (fever, cough, fatigue, etc.) during hospitalization. Twelve (50.0%) cases showed typical CT scan images of ground-glass chest and 5 (20.8%) presented stripe shadowing in the lungs. The remaining 7 (29.2%) cases had a normal CT image and had no symptoms during hospitalization and none of the 24 cases developed severe COVID-19 pneumonia. In this study, we report 8 incidentally detected cases of COVID-19 pneumonia in chest computed tomography (CT) scan of patients referred to emergency department following multiple trauma without any respiratory symptoms. 

This cases series study was performed on multiple trauma patients who were referred to emergency department of Imam Hossein Hospital, Tehran, Iran, from 17 to 28 March 2020. Patients who underwent chest CT scan for trauma management and had radiographic manifestations of COVID-19 pneumonia on CT scan were enrolled. Demographic information (age, gender), mechanism of trauma, as well as chest CT scan and RT-PCR for COVID-19 results were collected and reported for enrolled cases. 

## Case presentation:

**Figure 1:**
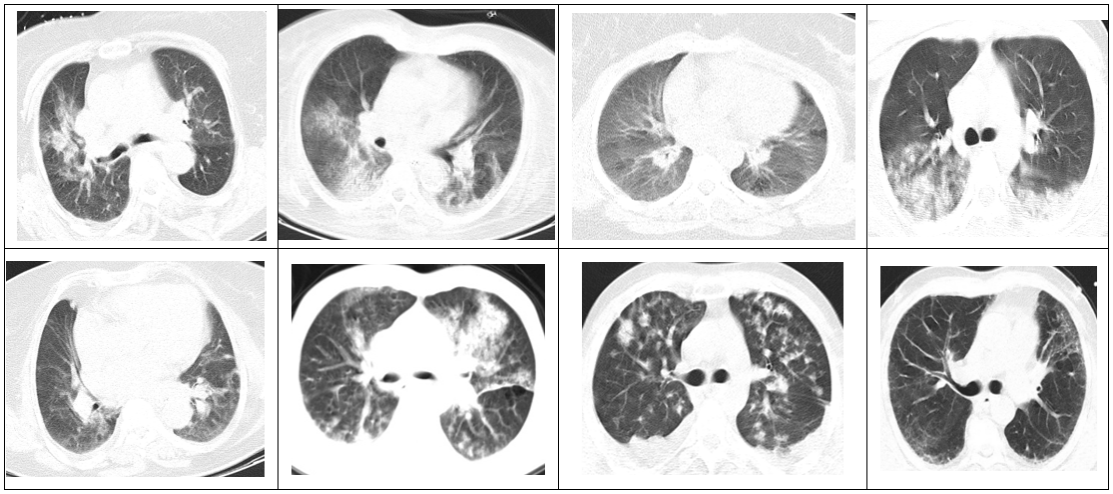
Axial Chest computed tomography (CT) scan without contrast of asymptomatic COVID-19 patients obtained during evaluation for multiple trauma.

 8 patients with the mean age of 49.71 ± 13.13 (range: 34 – 67) years were studied (62.5% male). The trauma mechanism was fall from height in 5 (62.5%) and car accident in 3 (37.5%) cases. None of the patients had symptoms in favor of COVID-19 infection such as fever, dyspnea, headache, cough, etc. at the time of admission. 5 (62.5%) patient had history of close contact with a suspected COVID-19 case. Physical examinations of lungs revealed no signs of pneumonia. A chest CT scan without contrast was performed to evaluate high-energy trauma and the findings strongly were in favor of pneumonia. The results of RT-PCR was positive for COVID-19 infection in all patients. During the hospital stay, two patients (25%) experienced mild symptoms such as fever, cough, and myalgia and the rest stayed asymptomatic. In the laboratory results, 4 (50%) patients had a slight increase in C - reactive protein (Maximum 18) and the rest of the laboratory findings were normal. Two patients underwent orthopedic surgery without any respiratory complication. All patients received standard treatment (Hydroxychloroquine with or without Azithromycin). None of the patients required intensive care. All patients were discharged from hospital after completion of treatment and had no mortality. Figure 1 shows axial cuts of the cases’ chest CT scan.

## Discussion:

The widespread distribution of COVID-19 is a major concern, globally. Understanding of the transmission risk is incomplete. Epidemiologic investigation in Wuhan at the beginning of the outbreak identified an initial association with a seafood market that sold live animals, where most patients had worked or visited, which was subsequently closed for disinfection (7). With droplet transmission, virus is shed via the respiratory secretions when a person with infection coughs, sneezes, or talks, which can infect another person if it makes direct contact with their mucous membranes. Infection can also occur if a person touches an infected surface and then touches his or her eyes, nose, or mouth. Droplets typically do not travel more than six feet (about two meters) and do not linger in the air (8). The interval during which an individual with COVID-19 is infectious is uncertain and the maximum incubation period for COVID-19 is thought to be 14 days following exposure, with most cases occurring approximately four to five days after exposure. During the incubation period, the patient can pass on the disease to others (9). In the study performed by Khazaee et al. (5), several patients had incidental evidence of COVID-19 infection on chest CT scan for trauma management. In the present study, all patients had evidence suggesting COVID-19 in CT scan, which was confirmed by RT-PCR after hospital admission. In this study, only 2 patients experienced mild symptoms and none of the patients developed severe symptoms. None of the patients in Hu et al. (6), study had severe illness and no death occurred. They observed a typical asymptomatic transmission to family members living in the same household, which even caused severe COVID-19 pneumonia. Overall, the asymptomatic carriers identified from close contacts were prone to becoming mildly ill during hospitalization. 

Physicians and therapists working in trauma centers should treat patients with extreme caution and personal protection. Also, during this pandemic, measures must be taken in trauma emergency departments to prevent transmission of the disease.

## Conclusion:

According to the findings of this study, asymptomatic trauma patients can be carriers of the disease and cause transmission. It is important for physicians and staff at trauma centers to know how to use personal protective equipment to prevent catching the disease.
